# Fenofibrate in the management of AbdoMinal aortic anEurysm (FAME): study protocol for a randomised controlled trial

**DOI:** 10.1186/s13063-016-1752-z

**Published:** 2017-01-04

**Authors:** Sophie E. Rowbotham, Doug Cavaye, Rene Jaeggi, Jason S. Jenkins, Corey S. Moran, Joseph V. Moxon, Jenna L. Pinchbeck, Frank Quigley, Christopher M. Reid, Jonathan Golledge

**Affiliations:** 1 The University of Queensland, School of Medicine, Herston, QLD 4006 Australia; 2 Department of Vascular Surgery, The Royal Brisbane and Women’s Hospital, Herston, QLD 4029 Australia; 3 Department of Vascular Surgery. Holy Spirit Northside Private Hospital, Chermside, QLD 4032 Australia; 4 Queensland Research Centre for Peripheral Vascular Disease, James Cook University, Townsville, QLD 4811 Australia; 5 College of Medicine and Dentistry, James Cook University, Townsville, QLD 4811 Australia; 6 Department of Vascular Surgery, The Royal Brisbane and Women’s Hospital, Herston, QLD 4029 Australia; 7 Mater Medical Centre, Pimlico, QLD 4812 Australia; 8 School of Public Health, Curtin University, Perth, WA 6000 Australia; 9 School of Public Health and Preventive Medicine, Monash University, Melbourne, VIC 3004 Australia; 10 Department of Vascular and Endovascular Surgery, The Townsville Hospital, Townsville, QLD 4811 Australia

**Keywords:** Abdominal aortic aneurysm, Fenofibrate, Clinical trial, Osteopontin, Macrophage

## Abstract

**Background:**

Abdominal aortic aneurysm (AAA) is a slowly progressive destructive process of the main abdominal artery. Experimental studies indicate that fibrates exert beneficial effects on AAAs by mechanisms involving both serum lipid modification and favourable changes to the AAA wall.

**Methods/design:**

Fenofibrate in the management of AbdoMinal aortic anEurysm (FAME) is a multicentre, randomised, double-blind, placebo-controlled clinical trial to assess the effect of orally administered therapy with fenofibrate on key pathological markers of AAA in patients undergoing open AAA repair. A total of 42 participants scheduled for an elective open AAA repair will be randomly assigned to either 145 mg of fenofibrate per day or identical placebo for a minimum period of 2 weeks prior to surgery. Primary outcome measures will be macrophage number and osteopontin (OPN) concentration within the AAA wall as well as serum concentrations of OPN. Secondary outcome measures will include levels of matrix metalloproteinases and proinflammatory cytokines within the AAA wall, periaortic fat and intramural thrombus and circulating concentrations of AAA biomarkers.

**Discussion:**

At present, there is no recognised medical therapy to limit AAA progression. The FAME trial aims to assess the ability of fenofibrate to alter tissue markers of AAA pathology.

**Trial registration:**

Australian New Zealand Clinical Trials Registry, ACTRN12612001226897. Registered on 20 November 2012.

**Electronic supplementary material:**

The online version of this article (doi:10.1186/s13063-016-1752-z) contains supplementary material, which is available to authorized users.

## Background

An abdominal aortic aneurysm (AAA) is defined as a progressive dilation of the infrarenal aorta, and is associated with a risk of fatal rupture which increases at larger AAA diameters [1, 2]. The incidence of AAA increases with advancing age, with approximately 5% of men and approximately 1% of women aged over 65 years having an AAA [3–7]. Other risk factors include smoking, hypertension, Caucasian ethnicity and a positive family history [8, 9]. Small AAAs (30–54 mm in diameter) are typically asymptomatic and may be detected as an incidental finding on imaging performed for other purposes, or as a pulsatile abdominal mass on routine physical examination. AAA screening programmes using ultrasound have been introduced in the United Kingdom, the United States and Sweden and are expected to reduce aneurysm-related mortality [10–13]. There is no recognised medical therapy for AAAs, with current management comprising regular ultrasound surveillance, until a diameter threshold is reached (typically 55 mm), at which point surgical repair is considered as the risk of aortic rupture is considered to be high for most patients [14].

Identification of an effective drug therapy to limit AAA progression would represent a significant advancement in clinical management. Clinical trials in humans have yet to report convincing benefit of any tested agent in slowing AAA growth [15–18]. However, preclinical studies continue to hold promise. Studies employing two rodent models reported that the peroxisome proliferator activator alpha (PPARα) ligand fenofibrate can reduce AAA development [19, 20]. Notably, in one study, fenofibrate-mediated protection against AAA formation was associated with the concomitant reduction of the proinflammatory protein osteopontin (OPN) and reduced recruitment of macrophages to the aortic wall [19]. Osteopontin (OPN) is a phosphorylated acidic glycoprotein that is implicated in many processes integral to AAA development including inflammation, proteolysis and atherosclerosis [21–26]. OPN deficiency has been shown to protect against AAA formation in angiotensin-II-infused apolipoprotein-e-deficient mice [27], and serum OPN has been shown to be independently associated with AAA presence and growth in humans [28]. Of significant importance to the development and progression of AAA in experimental models is the ability of OPN to promote macrophage accumulation within the aorta [27, 29]. Macrophages are implicated in aortic destruction as a result of the production of a range of proteolytic enzymes, such as matrix metalloproteinases (MMPs) [30], and marked macrophage infiltration is a consistent feature of human AAA [31].

PPARα ligands have been shown to downregulate OPN expression in human macrophages in vitro [32]. Fibrates are well-known PPARα ligands and are indicated in the treatment of hypertriglyceridemia [33]. Previous studies in rodent models suggest that fenofibrate downregulates OPN expression in hypertrophied left ventricle and dysfunctional renal cells [34, 35]. Furthermore, treatment of diabetic patients with bezafibrate for 4 weeks has been shown to reduce circulating concentrations of OPN by approximately 40% [32]. The ability of fenofibrate to downregulate OPN may be critical in reducing macrophage infiltration and the associated release of proteolytic enzymes, thus potentially limiting AAA expansion. Additionally, fenofibrate is known to elevate serum high-density lipoprotein (HDL) which has been associated with protection from AAA [31].

Collectively, the above findings lead to the hypothesis that a short course of fenofibrate will exert beneficial effects on AAA by mechanisms involving both serum lipid modification and favourable changes to the AAA wall. The aim of the current study is to assess the effect of fenofibrate taken daily for a minimum of 2 weeks in participants scheduled for elective open AAA repair. This group of patients is particularly suitable since the AAA will be replaced with a prosthetic graft enabling the AAA wall and thrombus to be removed for biological assessment. The primary aim of the study is to determine whether fenofibrate will reduce the relative number of AAA-wall macrophages, reduce the relative concentration of AAA-wall OPN and also reduce the serum concentrations of OPN. The effect of fenofibrate on secondary parameters, including inflammatory cell (neutrophils, B-and T-lymphocytes) number, MMPs and proinflammatory cytokines within the AAA wall, periaortic fat and intramural thrombus, and circulating concentrations of AAA biomarkers, including osteoprotegerin, resistin, D-dimer and fasting lipids, will also be assessed [31, 36–38].

## Methods/design

### Study design and participants

Fenofibrate in the management of AbdoMinal aortic anEurysm (FAME) is a multicentre, randomised, double-blind, placebo-controlled clinical trial to assess the effect of orally administered therapy with fenofibrate on key pathological markers of AAA in patients undergoing open AAA repair. The trial will be conducted at four sites in Australia: The Royal Brisbane and Women’s Hospital, Brisbane; The Holy Spirit Northside Private Hospital, Brisbane; The Townsville Hospital, Townsville and The Mater Hospital, Townsville. The trial will be reported according to the Standard Protocol Items: Recommendations for Intervention Trials (SPIRIT) (see Additional files 1 and 2). Only research personnel who are directly involved in the recruitment and data collection aspect of the study will have access to patients’ personal details. All Case Report Forms (CRFs), source documentation and samples will be stored de-identified where personal information has been removed and coded with a study number.

The FAME trial will include participants recruited from specialist vascular outpatient clinics who have an asymptomatic infrarenal AAA with a maximum orthogonal diameter ≥50 mm. FAME will not include individuals who require emergency or urgent AAA repair due to the requirement for a minimum 2 weeks of treatment with trial medication prior to surgery. Furthermore, participants will only be included if it is determined that they have a high likelihood of treatment compliance according to the treating physician or local study coordinator. Additional exclusion criteria include current treatment with fibrates, known contraindications to fenofibrate treatment and previous aortic surgery. A full list of inclusion and exclusion criteria is given in Table 1.

**Table 1:** Patient eligibility criteria

Inclusion criteria
• Ability to provide written informed consent • Diagnosis with an asymptomatic AAA which is infrarenal in location and measures ≥50 mm on CTA • Scheduled for an elective open repair of an AAA • High likelihood of medication compliance within the 2-week period prior to surgery
Exclusion criteria
• Currently taking fenofibrate or related fibrates • Contraindication to fenofibrate treatment: o Liver impairment as demonstrated by abnormal AST or ALT tests (>1.5 × ULN) o Renal impairment as demonstrated by an elevated serum creatinine level (>150 μM) o Symptomatic gallbladder disease o Previous reaction to any lipid-modifying medication • Previous infrarenal abdominal aortic surgery • Mycotic AAA • Requirement for emergency or urgent open AAA repair • Current participation in another drug trial

*CTA* computed tomographic angiography, *AAA* abdominal aortic aneurysm, *AST* aspartate aminotransferase, *ALT* alanine transaminase, *ULN* upper limit of normal

### Randomisation and follow-up

**Fig. 1:**
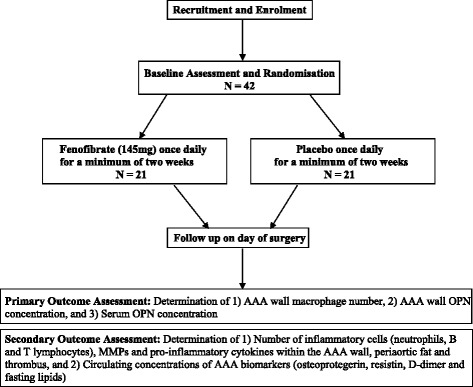
Schematic diagram of the Fenofibrate in the management of AbdoMinal aortic anEurysm (FAME) trial

The overall design of the FAME trial is shown in Fig. 1. At the initial visit, potential participants booked for an elective open repair of an AAA will be assessed against the eligibility criteria (Table 1) and, if appropriate, informed consent will be obtained. Individuals will undergo a medical examination, resting blood pressure and heart rate assessments, and collection of blood samples for measurement of full blood count (haemoglobin, white cell count, platelets, neutrophils, lymphocytes, monocytes, eosinophils, basophils), urea and electrolytes (sodium, potassium, creatinine, estimated glomerular filtration rate, urea, chloride, bicarbonate), liver function tests (Albumin, total bilirubin, alanine transaminase, aspartate aminotransferase, gamma-glutamyl transpeptidase, lactate dehydrogenase), fasting lipids (cholesterol, triglyceride, HDL cholesterol, low-density lipoprotein (LDL) cholesterol), fasting glucose and C-reactive protein. Serum, plasma and whole blood will also be collected for the later assessment of circulating concentrations of protein (such as cytokines) and genetic (deoxyribonucleic acid (DNA) and ribonucleic acid (RNA)) markers. Investigational blood samples will be collected into the following tubes: 2 × 5-mL SST, 2 × 4-mL EDTA tubes, 1 × 4-mL sodium citrate tube and 1 × 2.5-mL PaxGene tube. Blood samples will be processed according to site-specific standard operating procedures (SOPs) and shipped to the study centre in Townsville. Eligible participants will be randomised to receive 145 mg fenofibrate or placebo, administered once a day for a minimum of 2 weeks directly prior to surgery, in a parallel-group design. Randomisation to fenofibrate or placebo will be stratified by study centre. Random allocation sequences will be computer-generated by a statistician and provided to each study centre's clinical trial pharmacist, ensuring both investigators and participants are blinded to drug assignment. Trial medication will be allocated and dispensed by the local study centre’s clinical trials pharmacist. Allocation concealment will be achieved by using identical packaging of the intervention and placebo. In the case of an emergency situation where breaking of the group allocation blinding would be required, the local study centre clinical trial pharmacist will be contacted. To facilitate compliance, participants will be provided with the phone number of the local study coordinator with instructions to contact them in the event of possible medication-related problems or consideration of discontinuation. In this event arrangements will be made for the participant to be reviewed by the study physician to ascertain whether discontinuation is required.

On the day of surgery, further blood samples will be collected (as per initial visit). Adverse and clinical events will be recorded, changes to usual medication noted, and compliance with the study drug regimen analysed by capsule counting. During open surgery, biopsies will be taken from the following sites: (1) subcutaneous fat at the incision site, (2) periaortic fat near the AAA, (3) AAA neck, (4) AAA body (opposite the inferior mesenteric artery) and (5) AAA thrombus. To preserve RNA and protein integrity, tissue samples will be collected into liquid nitrogen immediately upon harvest for subsequent genetic and protein analysis. An additional AAA body sample will be collected and immediately stored in 10% (v/v) paraformaldehyde and wax-embedded for immunohistochemical analysis. All collection of tissues will be performed according to site-specific SOPs and shipped to the study centre in Townsville.

### Outcome assessment

Outcome assessment will be performed at the study centre in Townsville on the tissue and blood samples collected. All outcome assessment will be conducted by scientists blinded to the treatment allocation of the participants.

### Primary outcome assessment

To assess AAA-wall macrophage number, serial cryostat sections 7 μm thick will be cut from each AAA wall sample for subsequent macrophage staining, as previously described [19]. All samples will be stained simultaneously using identical reagents and incubation times. Serial frozen sections will be air-dried, fixed in acetone for 10 min at −20 °C, air-dried and rehydrated with phosphate-buffered saline (PBS) before being incubated in 3% H_2_O_2_/0.1% sodium azide/PBS to block endogenous peroxidase. For macrophage detection, sections will be blocked in 2% (v/v) normal goat serum in PBS followed by staining using pan-macrophage antibody (Abcam) and goat anti-rat HRP (Chemicon). An IgG (Sigma) will be used as isotype control. Slides will be incubated in the peroxidase substrate 3,3’-diaminobenzidine (ImmPACT DAB, Vector Laboratories), counterstained in Mayer’s haematoxylin, dehydrated, cleared in xylene and mounted in Depex mounting medium. Stained sections will be photographed using a Leica BMLB microscope fitted with a SPOTTM CCD Camera (Diagnostic Instruments, Inc., Sterling Heights, MI, USA) and digital images captured to a PC supported with SPOT32TM software (version 2.1.2; Diagnostic Instruments, Inc., Sterling Heights, MI, USA). Identical exposure times and settings will be used for sections. Image analysis will be performed on digital tiff images using Adobe Photoshop CS6 Extended software. For each section, the total tissue area and area of macrophage staining will be measured using the ‘Selection Tool’ and ‘Record Measurements’ functions. Macrophage staining will be expressed as macrophage number and also as a percentage of total tissue area.

AAA-wall OPN will be determined using an enzyme-linked immunosorbent assay (ELISA) and immunohistochemistry. For determination by ELISA, protein will be extracted from individual frozen AAA wall biopsies by homogenising in buffer (10 mM cacodylic acid, 60 mM L-arginine, 0.25% (v/v) Triton x-100 in PBS, pH 7.2) and centrifuging at 18,000 × g at 4 °C for 20 min. Supernatant protein will be quantified by the Bradford technique (Protein Assay, Bio-Rad, Hercules, CA, USA). OPN concentration will be measured by ELISA (Quantikine, R&D Systems for OPN) and expressed as pg/mg of protein. Excellent reproducibility of similar assays has previously been reported [39]. For determination by immunohistochemistry, slides will be incubated in 2% (v/v) normal goat serum (Vector Laboratories) in PBS and endogenous avidin and biotin-blocked using an Avidin/Biotin blocking kit (Vector Laboratories), then 2 μg/mL rabbit anti-human OPN (Immuno Biological Laboratories), biotinylated goat anti-rabbit IgG (Vector Laboratories) and Vectastain Elite ABC-HRP. Rabbit IgG (Vector Laboratories) will be used as isotype control antibody.

Serum OPN concentration will be measured using blood collected from participants following an overnight fast. Serum will be stored at −80 °C until later batch assessment using ELISA according to the manufacturer’s instructions, and expressed as ng/mL (R&D Systems). This assay has previously demonstrated excellent intra- and inter-assay reproducibility [39].

### Secondary outcome assessment

Additionally, inflammatory cells (neutrophils, B- and T-lymphocytes), MMPs and proinflammatory cytokines will be assessed by immunohistochemistry and ELISA as previously described [40, 41]. Circulating concentrations of other AAA biomarkers, including osteoprotegerin, resistin, D-dimer and fasting lipids, will be assessed by ELISA and automated assays as previously described [31, 36–38]. Whole genome microarrays and real-time polymerase chain reaction will be used to examine gene expression levels based on findings from ongoing expression arrays and biomarker studies.

### Study population and power calculation

Estimated outcomes for the control group are based on assessments performed in human AAA biopsies or blood samples from previous studies [28, 32, 42–44]. Estimated effect sizes for fenofibrate therapy are based on previous rodent and human studies, and the consideration of outcomes likely to be required for clinical efficacy [19, 28, 32, 42–44]. To significantly influence the natural history of aortic destruction, it is estimated that a reduction in all primary outcomes assessed within AAA biopsies of at least 50% is likely to be required. In a rodent model, 4 weeks’ treatment with fenofibrate reduced aortic OPN concentration and macrophage infiltration by a median of 95% and 70%, respectively, suggesting that this treatment effect size is realistic [19]. For serum OPN, it has previously been reported that a fibrate reduced circulating OPN concentration by approximately 40% after 4 weeks of treatment [32]. Based on these data, the estimated mean values for control and treatment groups were calculated and are given in Table 2. Sample sizes were calculated using GPower3.1, based on a *t* test as the statistical analysis test. Since there are three primary outcomes, alpha was set at .017, adjusted from .05 for multiple testing. Sample size was estimated based on a power of 80% and equal numbers of participants in each treatment arm. As a result, 20 participants receiving fenofibrate and 20 participants receiving placebo are required. Assuming a dropout rate of 5%, a total of 42 participants will be required.

**Table 2:** Summary of primary outcome measures and expected results

Primary outcome	Measurement method	Estimated outcomes	
		Placebo	Fenofibrate
AAA-wall OPN concentration (pg/mg)	ELISA	210 ± 145	84 ± 87
AAA-wall macrophages (per mm^2^ section)	IHC	4.9 ± 1.8	2.4 ± 1.8
Serum OPN concentration (ng/mL)	ELISA	77 ± 32	46 ± 32

*AAA* abdominal aortic aneurysm, *ELISA* enzyme-linked immunosorbent assay, *IHC* immunohistochemistry, *OPN* osteopontin

### Safety

Participant safety will be assessed prior to the administration of the medication and at the end of the study period. At the initial visit, a consultation with a physician will occur, during which the participant will be informed about known side effects including symptoms of abdominal/back pain, chest pain, and renal and liver dysfunction. Pathology tests consisting of full blood count, fasting lipids, glucose, inflammation markers, liver and renal function will be performed. The participant will undergo a physical assessment which will include blood pressure and heart rate measurements that will be reviewed by a physician along with the results of pathology tests prior to randomisation. At the final visit, pathology tests as per the initial visit will be performed and reviewed by a physician. Any adverse event will be reported to the coordinating centre and carefully monitored throughout the study. Serious adverse events (SAEs) will be defined as death, requirement for inpatient hospital treatment and persistent or significant disability. All SAEs will be reported by the site principle investigator to the HREC and reviewed by the chief principle investigator, where a decision regarding withdrawal of trial medication will be made. Where a decision to withdraw trial medication is made, participants will be encouraged to remain on the study protocol. Previous studies suggest that fenofibrate is a relatively safe medication [45, 46]. Participants who are concurrently on warfarin will have two additional safety assessments after randomisation. Current routine care for patients on warfarin involves ongoing measurement of International Normalised Ratio (INR) levels, which in turn dictates the dose of medication required to manage clotting without increasing the risk of excessive bleeding. Participants will be instructed to have their INR concentrations assessed via their usual system at 3–5 days and again at 14–21 days post first dose so that warfarin dosage may be adjusted accordingly.

### Data management and analysis

Trial documentation including protocols, SOPs and CRFs will be shared electronically with participating study centres. Protocol amendments will be submitted to the Royal Brisbane and Women’s Hospital HREC and local site research governance offices and disseminated to the relevant parties at each study site. Data recorded on printed CRFs will be scanned to the study centre in Townsville where it will be entered centrally and examined for data quality. This will allow confirmation of entry criteria and collection of set entry and outcome data. Examples of important baseline data which will be collected include age, gender, presence of diabetes and/or dyslipidaemia, concurrent medications and maximum aortic diameter. At completion of the trial the database will be checked for errors and data confirmed with source documentation where required. Analysis of primary and secondary endpoints will be based on intention-to-treat at the time of randomisation. All participants who meet the eligibility criteria, provide written informed consent and are enrolled in the study will be included in the primary analysis, regardless of adherence to medication allocation.

To identify potential confounders, collected clinical and demographic data will be compared between groups via univariate statistics. The distribution of all continuous data variables will be assessed for normality using the Kolgorov-Smirnov test. Normally distributed continuous variables will be compared between test groups via *t* test; non-normally continuous distributed variables will be compared between groups using the Mann-Whitney *U* test. Nominal data will be compared using the chi-squared test. Characteristics showing a *p* value < 0.100 on univariate tests will be considered as potential confounders and will be entered as covariates in subsequent binary logistic regression models assessing the association of each of the outcome measures with treatment allocation. Following binary logistic regression, the association of all covariates with treatment allocation will be reported as odds ratios and 95% confidence intervals. For all analyses, *p* values <0.05 will be considered to be significant. Data will be published in a peer-reviewed journal with copies of the paper available to participants if required.

## Discussion

The estimated global prevalence rate of AAA per 100,000 in 2010 has been reported to range from approximately 8 in individuals aged 40–44 years to approximately 2,275 in individuals aged 75–79 years [47]. Prevalence is reported to be higher in developed versus developing nations, and in 2010 was highest in Australasia [47]. The global death rate due to AAA per 100,000 has been reported to have increased from 2.49 in 1990 to 2.78 in 2010, with the highest mean death rate found to occur in Australasia [48]. At present there is no known effective medical therapy to limit AAA progression, and large randomised trials have failed to provide evidence that early elective endovascular repair (EVAR) or open surgery for patients with AAAs measuring 40–54 mm reduces mortality [49–52]. Current management of patients with small AAAs involves regular repeat imaging since most small AAAs slowly increase in size, with approximately 70% of 40–54-mm AAAs requiring surgical repair [49–53]. AAA surgery is associated with significant mortality (1–5%) and perioperative complications (approximately 20%) [50, 52, 54, 55]. Whilst EVAR has gained popularity in recent years due to reduced length of inpatient stay and reduced intensive care unit admissions, total hospital costs are significantly greater than those associated with open repair (approximately AU$23,000 versus approximately AU$18,500 for preoperative, operative, postoperative and 1-year follow-up costs) in part due to the requirement for lifelong surveillance and the high rate of need for reintervention [54].

In the current study the effect of a promising new medical therapy will be assessed to determine whether a short course of fenofibrate will inhibit AAA-OPN expression and associated macrophage-based inflammation, whilst inducing other potential beneficial effects such as raising HDL.

### Trial status

At the time of submission, recruitment was ongoing.

## Additional files

Additional file 1: SPIRIT 2013 Checklist. (DOC 122 kb)
Additional file 2: SPIRIT figure. Schedule of enrolment, interventions and assessments. (DOC 60 kb)

## Abbreviations

AAA: Abdominal aortic aneurysm
ALT: Alanine transaminase
AST: Aspartate aminotransferase
CRF: Case Report Form
CTA: Computed tomographic angiography
ELISA: Enzyme-linked immunosorbent assay
EVAR: Endovascular repair
FAME: Fenofibrate in the management of AbdoMinal aortic anEurysm
HDL: High-density lipoprotein
HREC: Human Research Ethics Committee
MMP: Matrix metalloproteinase
NHMRC: National Health and Medical Research Council
OPN: Osteopontin
PPARα: Peroxisome proliferator activator alpha
SAE: Serious adverse event
SOP: Standard operating procedure
SPIRIT: Standard Protocol Items: Recommendations for Intervention Trials
ULN: Upper limit of normal
