# Supplementary material for: Fenofibrate in the management of AbdoMinal aortic anEurysm (FAME): study protocol for a randomised controlled trial
Source: Trials. 2017 Jan 4;18:1. doi: 10.1186/s13063-016-1752-z (PMC5209849; doi:10.1186/s13063-016-1752-z)
Supplement: Additional file 2: — SPIRIT figure. Schedule of enrolment, interventions and assessments. (DOC 60 kb) [file 13063_2016_1752_MOESM2_ESM.doc]

SPIRIT figure. Schedule of enrolment, interventions, and assessments

|  | **STUDY PERIOD** | | | | |
| --- | --- | --- | --- | --- | --- |
|  | **Enrolment** | **Allocation** | **Post-allocation** | | **Close-out** |
| **TIMEPOINT*** | ***-t_1_*** | **0** | ***t_1_*** | ***t_2_*** | ***t_3_*** |
| **ENROLMENT:** |  |  |  |  |  |
| **Eligibility screen** | X |  |  |  |  |
| **Informed consent** | X |  |  |  |  |
| **Allocation** |  | X |  |  |  |
| **INTERVENTIONS:** |  |  |  |  |  |
| ***Fenofibrate*** |  |  |  |  |  |
| ***Placebo*** |  |  |  |  |  |
| **ASSESSMENTS:** |  |  |  |  |  |
| ***Medical examination*** | X |  |  |  |  |
| ***Resting blood pressure and heart rate*** | X |  |  |  |  |
| ***Collection of blood samples*** | X |  |  |  | X |
| ***AAA wall macrophage number*** |  |  |  |  | X |
| ***AAA wall OPN concentration*** |  |  |  |  | X |
| ***Serum OPN concentration*** | X |  |  |  | X |
| ***Inflammatory cells*** | X |  |  |  | X |
| ***MMPs*** | X |  |  |  | X |
| ***Pro-inflammatory cytokines*** | X |  |  |  | X |
| ***Osteoprotegerin*** | X |  |  |  | X |
| ***Resistin*** | X |  |  |  | X |
| ***D-dimer*** | X |  |  |  | X |
| ***Fasting lipids*** | X |  |  |  | X |

* 0 = Baseline, t_1_ = Day 1 of treatment, t_2_ = ≥ Day 14 of treatment, t_3_ = Day of surgery

AAA, abdominal aortic aneurysm; OPN, osteopontin; MMPs, matrix metalloproteinases.
